# HLA and non-HLA genes in Behçet’s disease: a multicentric study in the Spanish population

**DOI:** 10.1186/1479-5876-10-S3-P53

**Published:** 2012-11-28

**Authors:** Marco Antonio Montes-Cano, Marta Conde-Jaldon, Jose Raul Garcia-Lozano, Lourdes Ortiz-Fernandez, Maria Francisca Gonzalez-Escribano

**Affiliations:** 1 Dept. of Immunology, HU Virgen del Rocio, Sevilla, Spain

## Objective

The aims of this study were to further investigate the influence of the HLA region in Behçet´s disease (BD) and to explore the relationship with non-HLA genes recently described to be associated in other populations.

## Methods

A total of 208 BD patients and 283 ethnically matched controls were included in this study. HLA-A and HLA-B low resolution typing was carried out by PCR-SSOP Luminex. Eleven tag single nucleotide polymorphisms (SNPs) located outside of the HLA-region previously described associated with the disease in genome wide association studies with a minoritary allele frequency in Caucasian greater than 0.15 were genotyped using TaqMan assays.

## Results

Different HLA-A (A*24, A*31 and A*66) and HLA-B (B*51 and B*57) were associated to BD as risk factors, whereas others (A*03, B*35 and B*58) were found to be protective. Multivariate analysis performed grouping the HLA-A as well as the HLA-B risk and protective factors suggests 2-fold influence of HLA-B as a risk factor compared with HLA-A, but similiar influence of both loci as protective. When HLA risk factors were *considered* together, no influence of the HLA-A itself was detected. Regarding the non-HLA genes, *IL23R* was the most strongly associated to the disease in our population but its effect seems to be restricted to individuals bearing HLA-B risk factors.

## Conclusion

Different HLA-B as well as HLA-A are associated to BD in addition to HLA-B51. Other non-HLA genes, such as *IL23R*, play a role in the susceptibility of the disease, although its influence could be conditioned to the presence of HLA factors.

## Acknowledgements

Spanish Group for Behcet's disease study: Norberto Ortego-Centeno, María Jesús Castillo-Palma, Gerard Espinosa-Garriga, Miguel Angel González-Gay, Ana Celia Barnosi-Marín, Roser Solans, Patricia Fanlo, Teresa Camps, Santos Castañeda, Juan Sánchez-Bursón, Antonio Núñez-Roldán, Javier Martín, María Francisca González-Escribano.

This work was supported by Fondo de Investigaciones Sanitarias (FIS 10/1701), Fondos FEDER, Plan Andaluz de Investigación (PAI CTS-0197 and CTS-180), Red de Investigación Cooperativa en Enfermedades Inflamatorias y Reumáticas (RIER) and Consejería de Salud de la Junta de Andalucía (Exp. 0260/08).

